# In Vitro Hemostatic Activity of Novel Fish Gelatin–Alginate Sponge (FGAS) Prototype

**DOI:** 10.3390/polym16142047

**Published:** 2024-07-18

**Authors:** Heri Herliana, Harmas Yazid Yusuf, Avi Laviana, Ganesha Wandawa, Basril Abbas

**Affiliations:** 1 Doctoral Program, Faculty of Dentistry, Universitas Padjadjaran, Bandung 45124, Indonesia; 2 Department of Oral and Maxillofacial Surgery, Faculty of Dentistry, Universitas Padjadjaran, Bandung 45124, Indonesia; 3 Department of Orthodontics, Faculty of Dentistry, Universitas Padjadjaran, Bandung 45124, Indonesia; 4 The Indonesian Naval Dental Institute, Jakarta 10210, Indonesia; 5 Research Center for Radiation Process Technology, National Research and Innovation Agency (NRIA), Jakarta 12440, Indonesia

**Keywords:** biopolymers, hemostatic agent, blood coagulation

## Abstract

A hemostatic sponge prototype was successfully synthesized from fish gelatin as an alternative to mammalian gelatin; it was mixed with alginate in certain combinations, double cross-linked with calcium ions, and gamma irradiated at a dose of 20 kGy to improve the characteristics and effectiveness of its function as a local hemostatic agent. There were improvements in the physicochemical and mechanical properties, porosity index, absorption capacity, biodegradation properties, biocompatibility, and hemocompatibility of the fish gelatin–alginate sponge (FGAS) prototypes compared with the pure fish gelatin sponge. Hemostatic activity tests showed that the means for clotting time, prothrombin time, and activated partial thromboplastin time were shorter in the FGAS prototype than in the negative control, and there was no significant difference compared with the commercial gelatin sponge. The hemostatic mechanism of the FGAS prototype combined a passive mechanism as a concentrator factor and an active mechanism through the release of calcium ions as a coagulation factor in the coagulation cascade process.

## 1. Introduction

Uncontrolled bleeding is one of the leading causes of death in medical emergencies in civilian and military life. Approximately 80% of deaths in trauma cases in civilian society and 20% in the military are caused by exsanguination. Another cause is complications during surgical procedures, in which uncontrolled bleeding is the most common complication [1,2]. The risk of morbidity and mortality increases during surgical procedures, especially in cases of uncontrolled bleeding [3,4]. Despite the proper use of conventional techniques for hemorrhage control to avoid such complications when uncontrolled bleeding occurs, a broad range of hemostatic agents are available as adjunctive measures to enhance hemostasis [5]. To treat excessive bleeding in areas that are difficult to access with conventional methods, a variety of topical hemostatic agents including gelatin-based hemostatic agents are currently available on the market [6].

One of the most commonly used local hemostatic agents, especially after surgical procedures, is a mechanical hemostatic agent made from gelatin material in the shape of an absorbent sponge [7]. Gelatin is made from the denaturation of collagen-derived proteins through a limited thermos-hydrolysis process and is known as an essential natural biopolymer. It has the property to change shape reversibly between sol and gel [8,9]. The sources of gelatin as raw materials for biomaterials, including hemostatic sponges, are still dominated by mammalian gelatin from pigs and cows. Currently, the sources of gelatin used in the world originate from 46% pork skins, 29.4% cow skins, 23.1% beef bones, and 1.5% other sources such as poultry and fish [7,9].

Using gelatin from mammals such as pigs and cows is restricted because of religious and infectious disease concerns. Both Muslim and Jewish communities are prohibited from consuming pork-based products. Furthermore, some animal-borne infectious diseases, such as bovine spongiform encephalopathy (BSE) in cows and swine flu in pigs, pose a threat to human health [10,11]. Therefore, another source of gelatin, for example, fish gelatin, has recently been used as an alternative to mammalian gelatin.

Fish gelatin has been widely researched and developed as an alternative source to mammalian (cow and pig) gelatin. However, fish gelatin has shortcomings in terms of its physicochemical properties, mechanical strength, and gel stability compared with mammalian gelatin [12]. To improve the weak properties of fish gelatin, the mixing method can be used with other biopolymers, one of which is alginate, to form a gelatin–alginate composite compound. Alginate, which is found in many seaweeds, is also a natural polymer that is widely used in the biomedical field. Calcium alginate is one of the alginate salts that works effectively as a hemostatic agent. The fish gelatin–alginate composite can be further increased in its physicochemical properties using cross-linking methods, for example, by Ca^2+^ ionic cross-linking and gamma irradiation [13,14].

Some previous studies reported the advantages of using fish gelatin and alginate in medical, pharmaceutical, and food processing fields, including acceptance by all religious communities, lower-cost production, abundant sources, and no infectious disease transmission potency compared to mammalian gelatin [15,16]. This research aims to synthesize an FGAS prototype using blending methods and double cross-linking with Ca^2+^ ions and gamma irradiation. The next step is characterizing and testing the effectiveness of the FGAS prototype’s hemostatic function in the blood coagulation process.

## 2. Materials and Methods

### 2.1. Materials

Gelatin (Redman fish gelatin, food grade 200 bloom, and viscosity of 3.45 mPa^−s^) was purchased from Phoon Huat Pte. Ltd. (Singapore). Sodium alginate (analytical grade with a viscosity of 22 mPa^−s^) and calcium chloride (CaCl_2_) were purchased from Sigma-Aldrich Corporation (St. Louis, MA, USA), and commercial gelatin hemostatic sponges (Ceraspon) were purchased from PT. Swayasa Perkasa (Yogyakarta, Indonesia). The other reagents used in this study were at least analytically pure.

### 2.2. Methods

#### 2.2.1. Synthesis of the Fish Gelatin–Alginate Sponge (FGAS) Prototype

**Figure 1:**
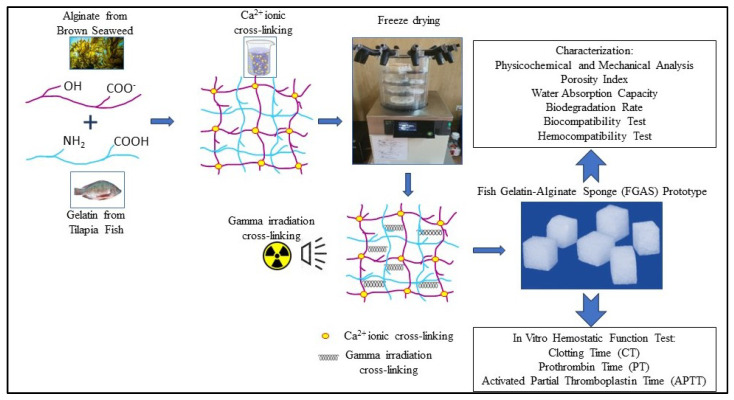
Diagrammatic representation of the research process.

First, fish gelatin and sodium alginate powder were blended in a beaker containing double-distilled water to obtain a 4% (*w*/*v*) mixed solution with certain proportions of fish gelatin (FG) and sodium alginate (SA) of 100/0 (wt%), 75:25 (wt%), 50:50 (wt%), and 25:75 (wt%). Then, the solutions were stirred evenly for 2 h at 50 °C, cast in a silicon mold to a size of 1 × 1 × 1 cm, and frozen at −20 °C. The frozen fish gelatin–alginate composites, except for the composition of 100:0 (pure fish gelatin), were then immersed in 2% CaCl_2_ to provide ionic cross-linking between the alginate and Ca^2+^ to produce a calcium alginate compound. In the next step, the Ca^2+^-cross-linked materials were then lyophilized with a freeze dryer at −50 °C for 24 h to obtain the fish gelatin–alginate sponge (FGAS) prototype. The final procedure was gamma irradiation cross-linking of the prototype materials at a 20 kGy dose—diagrammatic representation as shown in Figure 1 below. The prototype was prepared at the Research Center for Radiation Process Technology, Jakarta. All procedures were performed according to previous studies, with some modifications [17,18,19,20,21,22,23,24].

#### 2.2.2. Physicochemical and Mechanical Characterization

A scanning electron microscope (Thermo Fisher Scientific Phenom P-series, Eindhoven, The Netherland) was used to analyze the surface morphology of the prototype, followed by chemical element analysis using the energy-dispersive X-ray spectroscopy (EDX/EDS) method. The samples were additionally coated with a metal (e.g., gold or platinum) before examination to avoid sample charging and increased contrast when imaging under high vacuum conditions. The sample was inserted into the specimen chamber on the SEM–EDX machine for scanning at 300×, 500×, and 700× magnification. The electron source is emitted toward the sample to scan the sample surface, and then gold as a conductor reflects the electrons to the detector on the SEM–EDX microscope. The analysis of the FGAS prototype functional groups was performed using Fourier transform infrared spectroscopy (IRTracer-100, Shimadzu, Kyoto, Japan). All samples were prepared by grinding and mixing with KBr before being analyzed using an FTIR instrument with the ATR (Attenuated Total Reflectance) technique at wave numbers 400 cm^−1^–4000 cm^−1^. The mechanical properties were measured using the compressive strength test with the universal testing machine (Llyod Ametek, FL, USA) to determine the elasticity modulus of the FGAS prototype in wet conditions [25,26,27].

#### 2.2.3. Porosity Index Analysis

Porosity is an essential characteristic of sponge materials because it may directly impact the amount of water or blood they can absorb. There are various methods for measuring the porosity index of materials, including software like OriginPro ver-2023b and ImageJ 1.54i version [28,29,30]. We used OriginPro ver-2023b software to convert the images from SEM to quantitative measurements.

#### 2.2.4. Water Absorption Capacity

The swelling test is a standard procedure for analyzing the absorption capacity of materials. The dry sponge (W_0_) was weighed and placed in a pH 7.4 PBS solution for 30 min. Then, the sponge gel was wiped with filter paper to remove the residual solution from the sponge surface and weighed again (W_1_) [31]. The sponge’s water absorption rate was calculated using the following formula:

Water absorption rate (%) = (W_1_ − W_0_)/W_0_ × 100

#### 2.2.5. Biodegradation Rate

The dry sponge (W_0_) was weighed and placed in a pH 7.4 PBS solution at 37 °C for 1, 7, 14, and 30 days. On each day, the sponge was removed to dry, and then the sponge was weighed again (W_1_) [32]. The weight retention (%) was calculated using the following equation:

Weight retention (%) = (W_0_ − W_1_)/W_0_ × 100

#### 2.2.6. Biocompatibility (Cytotoxicity Test)

The cytotoxicity test was performed according to the international standard [33]. The sponges produced were incubated on BHK-21 fibroblasts for 24, 48, and 72 h to measure cell viability using the MTT (3-(4,5- dimethylthiazol-2-yl)-2,5-diphenyltetrazolium bromide) assay. Cell viability was measured by reading the optical density (OD) of the sample, control, and blank with an Elisa reader and converting it to the following formula:

Cell viability (%) = (OD_sample_ − OD_blank_)/(OD_sample_ − OD_blank_) × 100

#### 2.2.7. Hemocompatibility (Hemolysis Test)

Hemocompatibility is an essential criterion for any biomaterial that will interact closely with blood. The sponge samples were evaluated by hemolytic tests according to the international standard [34]. Each of these samples was mixed with 0.4 mL of the incubated blood sample individually. Normal saline and deionized water were used as negative (neg) and positive (pos) controls, respectively. After completing all of the hemolysis assay steps, all samples were centrifuged at 3000 rpm for 5 min. The absorbance of the supernatants was determined at 545 nm using a UV–Vis spectrophotometer and counted using the following equation:

Hemolysis rate (%) = (OD_sample_ − OD_neg control_)/(OD_pos control_ − OD_neg control_) × 100

#### 2.2.8. Clotting Time (CT)

The in vitro clotting time was determined using the previously described method. Each sponge sample was placed in a clean test tube. Then, the tubes were immersed in a water bath at a constant temperature of 37 °C for 1 h. Anticoagulated fresh donor blood (1 mL) was added to each of the test tubes and placed in the water bath. The tubes were tilted at 30° every 30 s until the blood in the tubes stopped flowing [23]. The coagulation times of blood in various tubes were recorded.

#### 2.2.9. Prothrombin Time (PT)

Prothrombin time (PT) is the in vitro clotting time test after placing the PT reagent, which contains thromboplastin (phospholipids with tissue factor), calcium, and citrated plasma [35]. A platelet-poor plasma (PPP) sample was obtained by centrifuging fresh human blood at 3000× *g* for 10 min. Each sponge sample (1.0 cm^3^) received 500 µL of PPP and was incubated for 30 min at 37 °C. To measure PT, 100 µL of PT reagent was combined with 200 µL of PPP in a test tube and incubated at 37 °C for 3 min. Then, PT was measured using an automated blood coagulation analyzer (COAX Bio System, Jerman). To serve as a control group, 500 µL of PPP extracted from fresh human blood was incubated at 37 °C for 30 min without any test material.

#### 2.2.10. Activated Partial Thromboplastin Time (APTT)

The APTT test measures the time it takes for plasma to clot in vitro after adding calcium, an activator of the intrinsic pathway, and the APTT reagent, which contains phospholipid, a platelet substitute that lacks tissue factor [35]. A platelet-poor plasma (PPP) sample was obtained by centrifuging fresh human blood at 3000× *g* for 10 min. Each sponge sample (1.0 cm^3^) received 500 µL of PPP and was incubated for 30 min at 37 °C. To measure APTT, 100 µL of PPP was incubated with 100 µL of reagent at 37 °C for 3 min. Finally, 100 mL of aqueous calcium chloride (CaCl_2_) was added to the solution, and the coagulation analyzer was used to determine the APTT value. To serve as a control group, 500 µL of PPP extracted from fresh human blood was incubated at 37 °C for 30 min without any test material.

### 2.3. Statistical Analysis

The experimental results are presented in terms of mean ± SD. The data were analyzed using the one-way ANOVA method for studies with more than two treatment groups, and the independent sample *t*-test method was used for studies with only two treatment groups.

## 3. Results and Discussion

### 3.1. Synthesis of the Fish Gelatin–Alginate Sponge (FGAS) Prototype

**Figure 2:**
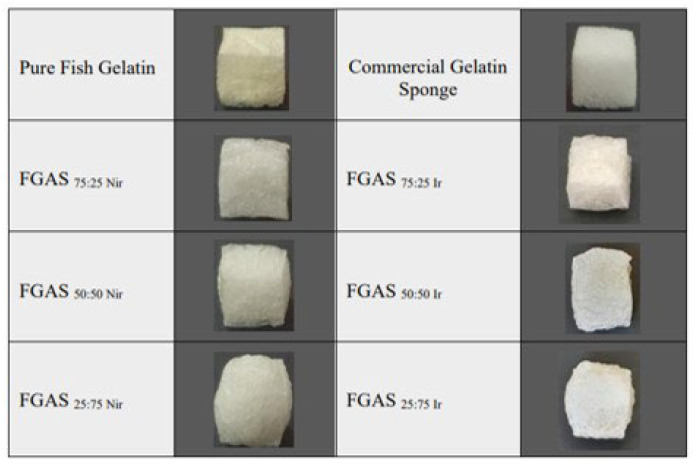
The FGAS prototype, as a result of the synthesis process, shows the various shapes of cuboid form in different material compositions.

Our research produced a fish gelatin–alginate sponge prototype, which was synthesized by mixing and freeze-drying methods from fish gelatin and sodium alginate with Ca^2+^ ions and gamma irradiation cross-linking, and was proven to have hemostatic characteristics and effectiveness against blood clotting. The FGAS prototype as shown in Figure 2, is a white sponge, cube-shaped, and has an interconnected porous structure containing chemical elements and functional groups combined from gelatin and alginate, as well as additional calcium ions.

At a larger gelatin composition (75:25), the sponge prototype material appeared cuboid with flat and straight edges. The addition of alginate compositions (50:50 and 25:75) changed the shape of the sponge prototype to become more rounded at each corner. This is thought to be caused by the increased alginate composition that resulted in more cross-linking between alginate and Ca^2+^ ions to form an “egg-box model” structure. This structure is formed by an ionic bond between the Ca^2+^ ions and the carboxyl group on the guluronic block of the alginate polymer chain, thereby forming a stable bond in the alginate gelation process [36,37]. The formation of more egg-box model structures due to increasing alginate concentration can cause shrinkage and compaction of the sponge material, starting at the weakest corner points, so that the sponge containing more alginate becomes rounded at the corners [38].

### 3.2. Physicochemical and Mechanical Characterization

**Figure 3:**
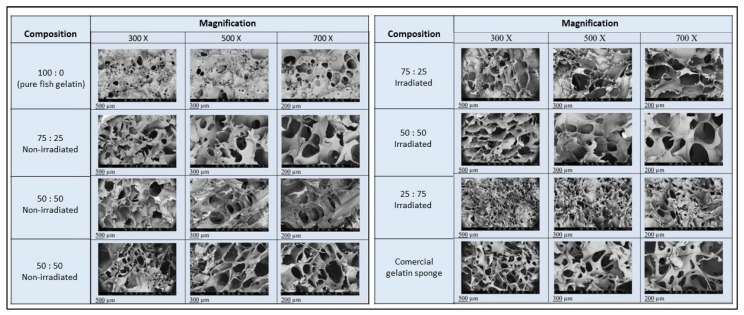
SEM examination of the surface morphology of the FGAS prototypes with magnifications of 300, 500, and 700× showing interconnected porous material.

The results of the SEM examination in Figure 3 show the surface morphology of the FGAS prototype, consisting of a porous structure with varying pore sizes, with an average size between 39.03 and 266.66 µm. As reported by previous research, these pore sizes qualify as hemostatic sponges, which states that a pore size of between 50 and 100 µm is sufficient to accommodate the concentration of red blood cells and platelets in the initial blood clotting process [39]. According to the IUPAC (International Union of Pure and Applied Chemistry) criteria, sponge material with a pore size of >50 µm is included in the macroporous category, which is ideal for containing cell concentrations because, in general, the average cell size is <100 µm [40].

Figure 3 above also shows the different characteristics between the samples according to various compositions of material and irradiation treatment. All fish gelatin–alginate composite sponge samples have more pores with sizes bigger than pure fish gelatin pore sizes. It is suspected that the addition of hydrophilic alginate binds more water, so that when the freeze-drying process is carried out, it leaves more pores. Meanwhile, the irradiated sample group shows denser pores and distributions caused by the cross-linking process.

The SEM images can also be used to analyze the chemical element content of the FGAS prototype combined with energy-dispersive X-ray spectroscopy (EDX/EDS) analysis, as shown in Table 1. The main chemical element composition of the FGAS prototype material consists of carbon (C), nitrogen (N), oxygen (O), and calcium (Ca). The elemental content corresponds to the main elements contained in the raw materials, namely fish gelatin and alginate, which consist of C, N, O, and H. The H element cannot be detected using EDX analysis because it is very light. Calcium (Ca) is an additional element formed by cross-linking sodium alginate with Ca^2+^ ions in the CaCl_2_ solution [41].

Table 1 above shows that the chemical elements composition in the irradiated sample group is greater than in the non-irradiated sample group. It is suspected that gamma irradiation caused the radiolysis of water and generated more free radicals; therefore, the chemical elements composition increased [42]. 

**Figure 4:**
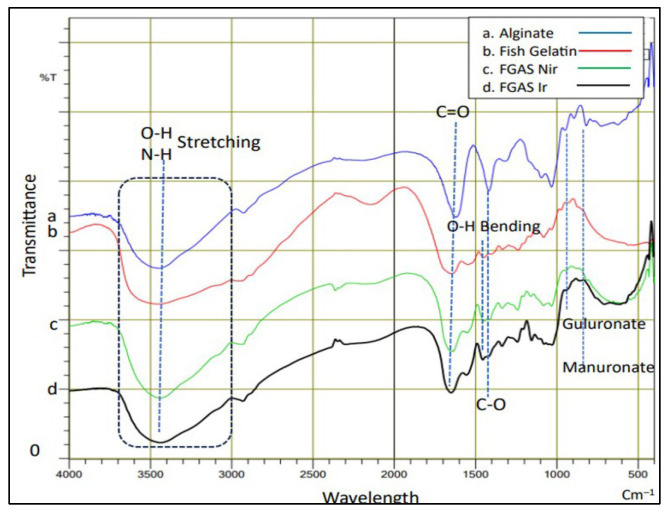
FTIR spectra of alginate, gelatin, and the FGAS prototypes.

The analysis of the FGAS prototype functional groups was carried out using the Fourier transform infrared (FTIR) test to observe the wavelength spectrum in the form of specific peaks that indicate the functional groups possessed by the material, as shown in Figure 4 below.

Figure 4 shows the peaks of the infrared light absorption spectra for typical functional groups found in gelatin polymers, alginate, and gelatin–alginate composites. Gelatin includes hydroxyl groups (O-H) and amine groups (N-H) at peak 3435.28 cm^−1^ (superimposed) and carbonyl (C=O) at 1653.99 cm^−1^. Alginate includes hydroxyl (O-H) at 3439.14 cm^−1^; carbonyl (C=O) at 1621.20 cm^−1^; carboxyl (C-O) at 1314.51 cm^−1^; guluronate and mannuronate fingerprints at 894.99 cm^−1^ and 819.76 cm^−1^, respectively. The FTIR spectrum for the gelatin–alginate composite is a combination of the spectrum of functional groups from gelatin and alginate polymers. The effect of gamma radiation can be seen from the shift in the absorption peaks of functional groups toward larger wavelengths in the group of samples that received gamma irradiation. The water radiolysis process caused by gamma irradiation generates free radicals to ionize carboxyl groups, and the ionized carboxyl groups react with amine groups to form amides. This process caused changes in the absorption peaks of the sample’s functional group in the FTIR spectrum. These findings confirmed the previous studies of Hariyanti et al., Perkasa et al., and Derkach et al. [20,42,43].

**Figure 5:**
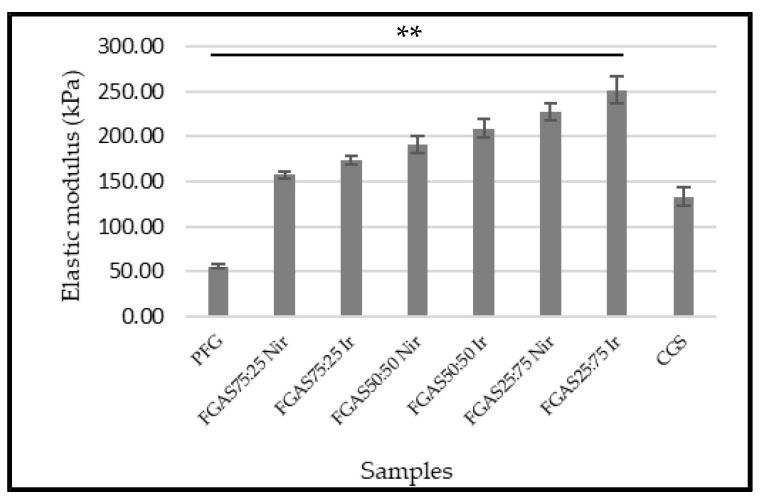
Elastic moduli of the FGAS prototypes in wet conditions (** *p* < 0.01).

The compressive strength test is a method for determining the mechanical properties of a biomaterial by measuring the elastic modulus, which is an important parameter in analyzing the characteristics of a sponge-shaped biomaterial that will be applied as a local hemostatic agent [44]. The sponge elastic modulus measurement was performed in wet conditions according to the purpose of hemostatic sponge application in bleeding situations, as shown in Figure 5 below.

All sponge samples showed greater elasticity modulus means compared with PFGS and were statistically significant. The FGAS_75:25 Nir_ and FGAS_75:25 Ir_ (157.79 ± 3.99 and 173.86 ± 5.18, respectively) have the closest elastic modulus values to commercial gelatin sponges (133.54 ± 9.79), but there was no significant difference in the elastic modulus between irradiated and non-irradiated samples. Fish gelatin has low mechanical strength, especially in a single form; therefore, modifications are needed both in the synthesis method and by adding reinforcement materials to increase this strength [45,46].

The increase in the elastic modulus of the FGAS prototype is thought to be due to the addition of alginate, increasing density and shrinkage of the polymer chain caused by the formation of an egg-box model structure due to the ionic cross-linking reaction between the guluronate residue of the alginate polymer and the divalent Ca^2+^ cation. As reported by Ma et al., in the preparation of fish gelatin hydrogel mixed with alginate, there was an increase in the elastic modulus [16].

### 3.3. Porosity Index Analysis

**Figure 6:**
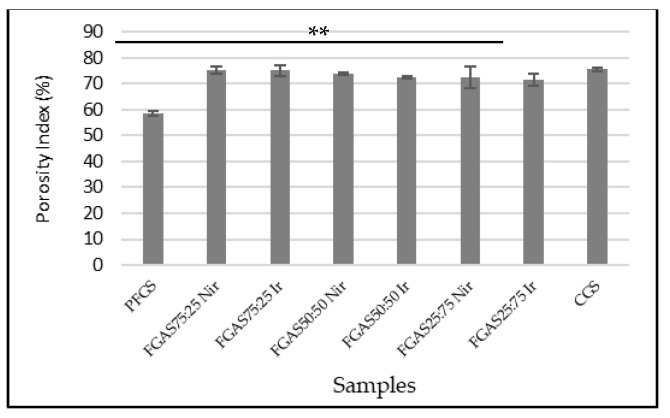
Porosity indices of FGAS prototypes (** *p* < 0.01).

Porosity is an essential characteristic of sponge materials because it may directly impact the amount of water or blood they can absorb, and this characteristic is strongly reliant on the underlying structure and morphology of sponges [28,29]. We analyzed the porosity index using OriginPro ver-2023b software, and the results are shown in Figure 6. All FGAS samples showed greater porosity index means compared with PFGS and were statistically significant.

The addition of alginate increases the porosity index of the FGAS prototype above the standard value of 60% for all FGAS prototype compositions, both irradiated and non-irradiated, namely between 71.67% and 75.32%. The porosity indices of FGAS_75:25 Nir_ and FGAS_75:25 Ir_ have the largest means (75.33% ± 1.25 and 75.07% ± 2.15) compared to all samples and show no significant difference compared to CGS. This is thought to be due to the addition of alginate, which is hydrophilic and binds water more strongly than fish gelatin during mixing so that when the lyophilization process (freeze-drying) is performed, it leaves more pores in the FGAS prototype material [47,48]. Sponge’s highly porous nature makes it easier for it to absorb blood fluid, which in turn minimizes the amount of excess exudate. Similarly, the spongy hemostatic agents and wound dressings rely on the sponge’s absorption properties, which depend on the shape and structure of the sponges [28,29,30]. The comparison between irradiated and non-irradiated samples shows that there was no significant difference in the porosity index.

### 3.4. Water Absorption Capacity

**Figure 7:**
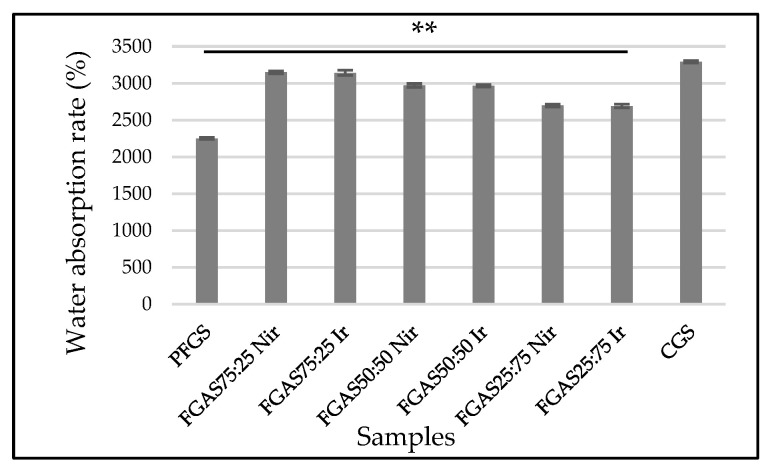
Water absorption capacities of FGAS prototypes (** *p* < 0.01).

Absorbing fluid or blood is one of the main requirements for materials with high absorption capacity such as local hemostatic sponges [31]. The results of the water absorption rate of the FGAS prototype are shown in Figure 7 below, where all FGAS samples show greater water absorption rates than PFGS and are statistically significant. 

The addition of alginate can increase the absorption capacity ratio of the FGAS prototype. As seen in Figure 7 above, the FGAS_75:25 Nir_ and FGAS_75:25 Ir_ prototypes have the largest mean absorption capacities of 3147.45 ± 19.87% and 3141.58 ± 34.22%, respectively, and are significantly different statistically compared to the mean absorption capacity of PFGS. This is possible because of the addition of alginate, which has greater hydrophilic properties, so it can bind more water molecules. During the lyophilization process, the bound water molecules will be sublimated, leaving many spaces or pores in the material [49]. There was no significant difference in the water absorption capacity between the irradiated and non-irradiated samples.

### 3.5. Biodegradation Rate

**Figure 8:**
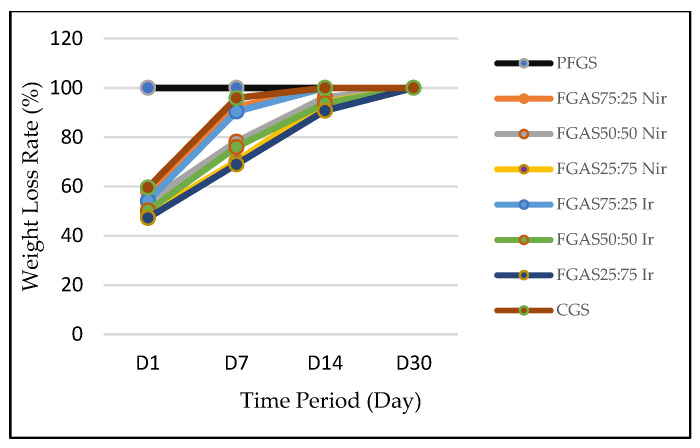
Biodegradation rates of FGAS prototypes.

The biodegradation rate of the FGAS prototype can be measured by counting the weight retention after immersing the sponge in a solution for a certain period [32]. The results of the biodegradation rate of the FGAS prototype are shown in Figure 8, where all sponge samples showed slower degradation rates compared with PFGS.

On Day 1 of observation, the FGAS prototype group, both irradiated and non-irradiated, showed a smaller weight loss ratio compared with PFGS, which was completely dissolved (100%) and did not qualify as a hemostatic sponge. The rapid degradation of pure fish gelatin sponge (PFGS) is thought to be due to the amino acid hydroxyproline content in fish gelatin being lower than that of mammalian gelatin, causing a low solubility (melting point) at a temperature of 25 °C–27 °C compared with mammalian gelatin at 32 °C–35 °C [50]. This was confirmed by Yang et al., who reported that pure gelatin sponges without cross-linking can immediately dissolve when immersed in PBS solution [51].

The FGAS prototype has a slower biodegradation ratio than PGFS, allegedly due to the addition of alginate and the presence of ionic cross-linking with Ca^2+^ to form calcium alginate compounds, which are difficult to dissolve in water, plus the combination of hydrogen bonds between gelatin and alginate molecules, which are strengthened by covalent bonds through cross-linking by gamma irradiation [32,52]. On Days 7, 14, and 30, it was shown that FGAS_75:25 Nir_ and FGAS_75:25 Ir_ have similar weight loss rates to CGS. There was also a significant difference in biodegradation rates between irradiated and non-irradiated FGAS samples. This proves that gamma irradiation can strengthen the bonds between peptide molecules in gelatin and between functional groups of gelatin and alginate through covalent bonds [53,54].

### 3.6. Biocompatibility (Cytotoxicity Test)

**Figure 9:**
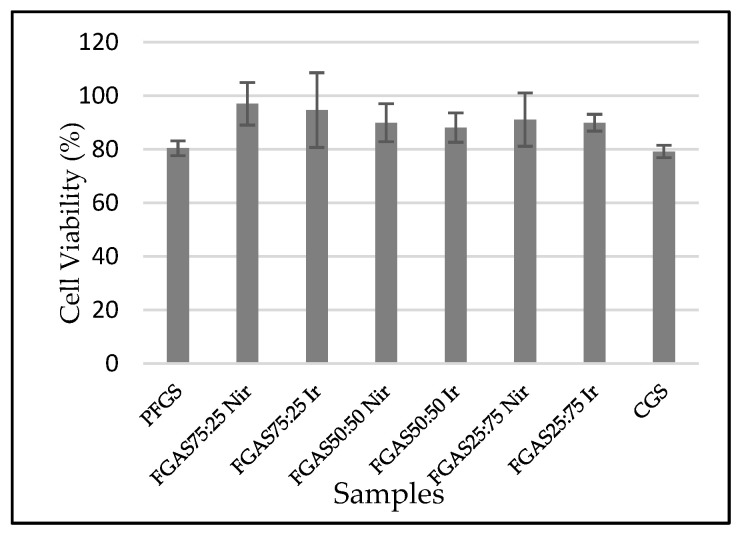
Cytotoxicity test results of FGAS prototypes.

Based on the MTT assay test results presented in Figure 9, it can be seen that all samples show an average viability of above 70% and no significant differences, which means that all samples meet the biocompatibility requirements according to the international standard [55].

The cytotoxicity test results prove that all FGAS prototypes, both irradiated and non-irradiated, have non-cytotoxic properties. Statistical testing using one-way ANOVA revealed no significant differences in the mean cell viability values for all research samples. These results confirmed those of several previous studies, such as Rezaie et al., who synthesized a highly absorbent sponge from bovine gelatin and sodium alginate by cross-linking using CaCl_2_. The cytotoxicity test results on human fibroblast cells showed that the viability of all test samples was above 80% [18]. Another study reporting the effect of gamma irradiation at a dose of 25 kGy on the cytotoxicity of gelatin–alginate bioadhesive material showed that the average overall fibroblast cell viability value was 89–100% [53].

### 3.7. Hemolysis Test

**Figure 10:**
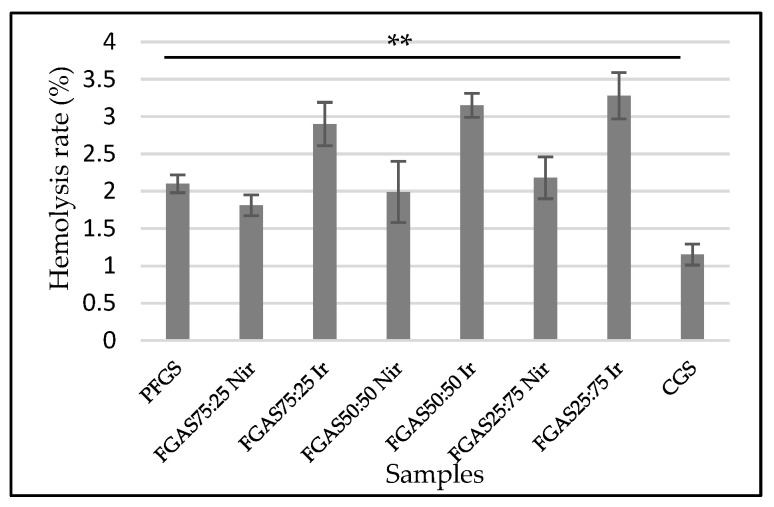
Hemocompatibility rates of FGAS prototypes (** *p* < 0.01).

The hemolysis test results, as presented in Figure 10, show that all samples show significant differences in erythrocyte hemolysis rates but are still below 5%, which means that all samples met the hemocompatibility requirements according to the international standard [34].

There was also a significant difference in hemolysis rates between the irradiated and nonirradiated FGAS samples. These findings confirmed the results of previous research by Rallapalli et al., who reported the effect of gamma irradiation at a dose of 25 kGy as a sterilization method for bovine pericardium scaffolds on the hemolysis ratio. There was an increase in the average hemolysis ratio from 1.8% before irradiation to 6.32% after irradiation. The increase in the hemolysis ratio is considered due to the irradiation process causing damage to the surface of the biomaterial, which becomes rougher so that it can damage erythrocytes and result in the lysis of hemoglobin [56].

Hemocompatibility characteristics are important for assessing interactions between drugs or biomaterials that function or are related to the circulatory system. Hemocompatible drug compositions or materials are capable of interacting with blood components without causing clinically significant adverse reactions such as thrombosis, hemolysis, complement activation, or other adverse side effects [57].

### 3.8. Hemostatic Activity Test

At this stage, the hemostatic function effectiveness test was only performed on the best FGAS prototype, which resulted from the characterization test in the previous stage where the FGAS_75:25 Nir_ and FGAS_75:25 Ir_ were found to be the selected prototypes.

#### 3.8.1. Clotting Time (CT)

**Figure 11:**
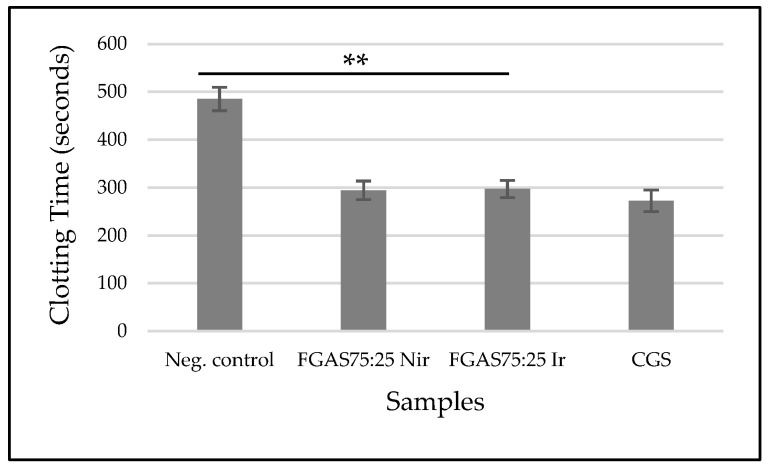
Clotting times of FGAS prototypes (** *p* < 0.01).

Based on the results of the blood clotting time test, as shown in Figure 11 below, the mean clotting time for the FGAS_75:25 Nir_ prototype was 294.33 ± 24.36 s and the FGAS_75:25 Ir_ prototype was 297.17 ± 19 s; these are significantly different compared with the negative control (485.00 ± 24.36 s), but not significantly different from CGS (272.33 ± 22.47 s). There was no significant difference in clotting time between the irradiated and non-irradiated samples. 

**Figure 12:**
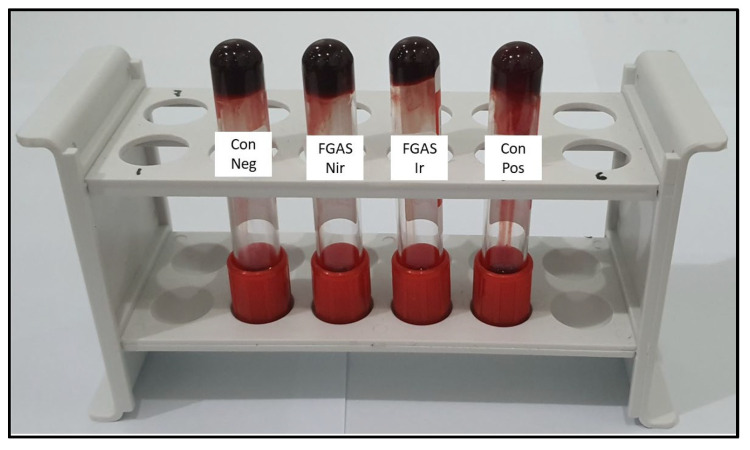
Clot-formed blood in the clotting time test. Con Neg: negative control (clot without any treatment), FGAS Nir: fish gelatin–alginate sponge non-irradiated, FGAS Ir: fish gelatin–alginate sponge irradiated, Con Pos: positive control (commercial gelatin sponge).

The FGAS prototype has an effective function as a local hemostatic agent by accelerating the bleeding time due to the porous structure of the hemostatic sponge, which allows it to absorb a lot of blood fluid while concentrating coagulation components, red blood cells, and platelets. Immediately upon contact with blood, the hemostatic sponge causes adhesion and aggregation of platelets, which in turn causes the formation of a platelet plug, thereby preventing blood flow from the wound. It also triggers the release of blood clotting factors involved in the extrinsic and intrinsic coagulation pathways, resulting in formation of a stable blood clot that helps control bleeding from wounds, as shown in Figure 12 [39].

Several previous studies have confirmed the use of gelatin and alginate as local hemostatic agents, including research by Dai et al., who reported that the manufacture of gelatin (GA), calcium alginate (CA), and silk fibroin (SF) composite sponges had good effectiveness by speeding up blood clotting time [58]. Chen et al. reported that the synthesis of gelatin–alginate hemostatic sponge with the addition of curcumin was effective in accelerating blood clotting time and preventing tumor recurrence [32].

#### 3.8.2. Prothrombin Time (PT)

**Figure 13:**
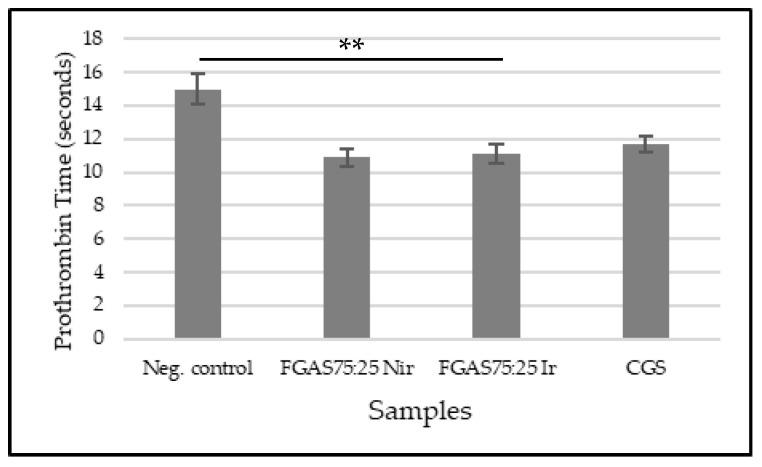
Prothrombin times of FGAS prototypes (** *p* < 0.01).

Figure 13 shows that the FGAS_75:25 Nir_ and FGAS_75:25 Ir_ prototypes have the shortest mean prothrombin times, 10.9 ± 0.52 and 11.1 ± 0.59 s, respectively, which are significantly different compared with the negative control (15.0 ± 0.87 s) but not significantly different from CGS (11.7 ± 0.48 s). There was also no significant difference in prothrombin time between the irradiated and non-irradiated samples. 

The effectiveness of the FGAS prototype in accelerating prothrombin time (PT) is considered due to the content of calcium ions bound in alginate. When in contact with blood, the FGAS prototype turns into a hydrogel, and a reaction occurs that encourages the entry of calcium ions into the wound through an ion exchange reaction with sodium ions in the blood. Furthermore, calcium ions trigger the production of coagulation factors VII, IX, and X, as well as platelets, activate the coagulation cascade reaction, and accelerate the hemostasis process [59].

Previous research has reported the role of calcium alginate in the coagulation process because it contains phytohemagglutinin, which can trigger red blood cell aggregation and change erythrocyte morphology, exposing phosphatidylserine on the surface of erythrocytes and accelerating the conversion of local prothrombin to thrombin [60]. Dai et al. reported the hemostatic effectiveness of a composite sponge made from silk fibroin, gelatin, and calcium alginate by accelerating prothrombin time [58].

#### 3.8.3. Activated Partial Thromboplastin Time (APTT)

**Figure 14:**
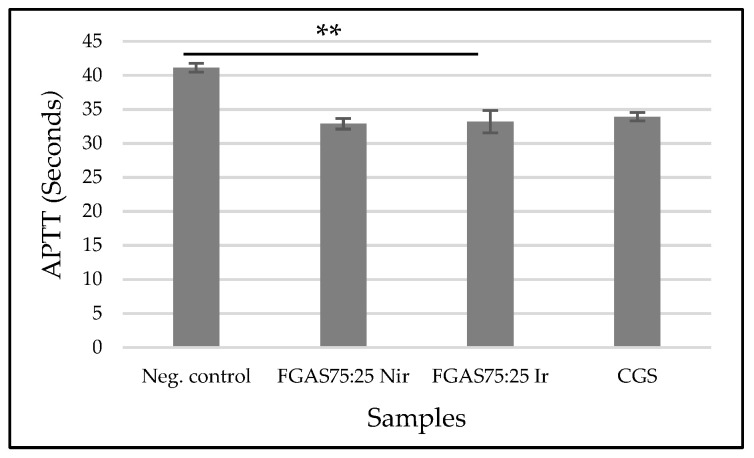
Activated partial thromboplastin times of FGAS prototypes (** *p* < 0.01).

The results of the APTT test, as shown in Figure 14, show that the FGAS_75:25 Nir_ and FGAS_75:25 Ir_ prototypes have the shortest APPT means, namely 32.88 ± 0.78 and 33.18 ± 1.64 s, respectively, which is significantly different from the negative control (41.12 ± 0.63), but there is no significant difference with CGS (33.92 ± 0.63). There was also no significant difference in the APTT means between the irradiated and non-irradiated samples.

Similar to the PT test, the APTT test results also suggest that the ability of the FGAS prototype to accelerate APTT was due to the calcium ion content bound in the alginate that was released when the sponge came into contact with the blood and then promoted the cascade coagulation process [60].

As reported by Che et al., in making a polyelectrolyte multilayer film on a sodium alginate/gelatin sponge, it has the effectiveness of shortening APTT, which is thought to be due to the activity of the calcium ions contained in the sponge [61]. Another study by Kumar et al. stated that the synthesis of calcium alginate–zinc chloride hydrogel is determined by the content of calcium ions, which can trigger clotting factors in the coagulation cascade process [62].

## 4. Conclusions

The FGAS prototype has better physicochemical and mechanical characteristics, porosity index, water absorption capacity, biodegradation rate, biocompatibility, and hemocompatibility than PFGS, and the FGAS_75:25Nir_ and FGAS_75:25Ir_ prototypes are selected as the best compositions. It is also proven that the FGAS prototype is effective as a local hemostatic agent, where the hemostatic mechanism is a combination of a passive mechanism as a concentrator factor through superiority in porosity index and absorption capacity, functioning as a matrix to collect blood cells, especially platelets, and coagulation factors; and it is also the active mechanism through the calcium ions (Ca^2+^), is released in the coagulation cascade process. The 20 kGy gamma irradiation dose only affected the biodegradation and hemolysis rate characteristics of the FGAS prototype.

## Figures and Tables

**Table 1:** Chemical elements compositions of the FGAS prototypes.

Sample	Composition		Chemical Elements (wt%)		
		Carbon (C)	Nitrogen (N)	Oxygen (O)	Calcium (Ca)
1	PFGS	22.478	21.578	16.583	-
2	FGAS_75:25 Nir_	5.506	12.613	10.711	30.030
3	FGAS_50:50 Nir_	6.300	4.600	9.800	25.600
4	FGAS_25:75 Nir_	1.800	1.900	5.300	18.700
5	FGAS_75:25 Ir_	20.500	27.000	8.900	22.000
6	FGAS_50:50 Ir_	18.418	15.516	14.715	19.119
7	FGAS_25:75 Ir_	17.700	6.900	16.700	17.700
8	CGS	35.200	33.900	30.900	-

PFGS: pure fish gelatin sponge, FGAS: fish gelatin-alginate sponge, Nir: non-irradiated, Ir: irradiated, CGS: commercial gelatin sponge.

## Data Availability

This article includes all data presented or analyzed during the study.
